# Editorial: Muscle and Tendon Plasticity and Interaction in Physiological and Pathological Conditions

**DOI:** 10.3389/fphys.2021.678801

**Published:** 2021-04-21

**Authors:** Falk Mersmann, Sebastian Bohm, Adamantios Arampatzis, Kiros Karamanidis, Olivier Seynnes

**Affiliations:** ^1^Department of Training and Movement Sciences, Humboldt-Universität zu Berlin, Berlin, Germany; ^2^Berlin School of Movement Science, Humboldt-Universität zu Berlin, Berlin, Germany; ^3^Sport and Exercise Science Research Centre, School of Applied Sciences, London South Bank University, London, United Kingdom; ^4^Department of Physical Performance, Norwegian School of Sport Sciences, Oslo, Norway

**Keywords:** adaptation, muscle contraction, strain energy, loading, maturation, aging, disease, injury risk

The interaction of muscle and tendon during movement is a crucial element for the performance of musculoskeletal systems. As both muscle and tendon are mechanosensitive and adapt to their mechanical environment, mechanical loading can introduce cellular responses affecting the properties of these tissues and the functional interplay between them. However, aging and pathology affect the muscle-tendon unit (MTU) in various ways, with direct consequences on its functional capacities. This Research Topic of Frontiers in Physiology focuses on current research considering muscle and tendon plasticity and their interaction during movement in physiological and pathological conditions.

It has been long recognized that the energy exchange within the MTU affects the operating conditions of muscle fibers and, thus, human performance during running and jumping. In their contribution, Hollville et al. demonstrated that the fascicle behavior of the vastus lateralis and gastrocnemius medialis muscle during countermovement jumping is not affected by the compliance of common sport surfaces (i.e., artificial turf, hybrid turf, and athletic track), while they recognized the possible role of the nervous systems to regulate muscular behavior to compensate for the differences in surface properties. Sano et al. showed for the first time that the interaction of muscle and tendon is also relevant during human swimming. As for terrestrial locomotion, the tendinous tissue of the quadriceps muscles seems to uncouple muscle and MTU strain, store and release energy and, thus, decrease the fascicle motion of the vastus lateralis muscle during a dolphin-kick. Moreover, the authors observed an increasing contribution of the tendinous tissue to MTU lengthening and shortening at higher swimming speed. Changes in tendon lengthening are predominantly governed by the magnitude of muscle force exertion, as suggested by the findings of Rosario and Roberts from a rat model, where loading rate had little influence on tendon fascicle mechanics. Despite tendon viscoelasticity, effects of loading rate on muscle fascicle behavior may only be relevant in MTUs with extremely high tendon-to-fiber length ratios, as observed in certain animals. Other contributions to this Research Topic also looked at intramuscular mechanisms that affect the operating conditions of its fibers. Konow et al. demonstrated that the shape-change of the rat gastrocnemius medialis muscle during contractions is tuned to the task requirements. Muscle bulging in the width direction was predominantly observed at high forces and may favor whole muscle force generation, while the greater bulging in thickness at low forces may facilitate muscle shortening. The understanding of muscle and tendon mechanics also progresses in research on injury and disease. In this article collection, Agres et al. showed that lengthening of gastrocnemius medialis fascicles is limited during passive ankle dorsiflexions, following Achilles tendon rupture, probably due to a longer and more compliant tendon. Moreover, this altered behavior does not seem to change with measures that primarily focus on the rehabilitation of independent gait. Tendons are also affected following muscle injury, as Barin et al. showed in a rat model, providing evidence of substantial extracellular matrix remodeling and changes in mechanical properties of the calcaneal tendon. Some of our contributors also examined the effects of pathologies affecting MTU function. Hösl et al. reported that children with cerebral palsy have lower gastrocnemius medialis muscle thickness and shorter fascicle compared with healthy peers, which directly associated with walking speed. During walking, the fascicles operated at shorter length, even normalized to resting length, and with lower velocities. Potential approaches for therapy are discussed by Kalkman et al. Their comprehensive review elaborated why stretching does not improve muscle morphology and function in children with cerebral palsy, and discusses alternative approaches such as combined therapies, eccentric training, or interventions for increasing tendon stiffness.

Across the human lifespan, both muscle and tendon adapt to changes of their mechanical environment. However, the degree of tissue plasticity may change with maturation and aging and muscles and tendons may differ considering their responsiveness to certain types of loading. Pentidis et al. reported that preadolescent artistic gymnastic athletes show higher muscle strength when normalized to body mass and jump performance, in spite of similar Achilles tendon stiffness compared to controls. In early and late adolescents, Charcharis et al. observed greater quadriceps femoris muscle strength and patellar tendon stiffness compared to untrained peers, although higher tendon strains were more prevalent in trained adolescents, which indicates an increased mechanical demand for the tendon. The data further suggests an increased risk for imbalances of muscle strength and tendon stiffness until adulthood. Mersmann et al. showed that in adolescent basketball athletes, high levels of tendon strain are associated with impairments of proximal tendon micromorphology similar to those observed with tendinopathy. In adult middle-distance runners, Devaprakash et al. found both higher plantar flexor muscle strength and Achilles tendon stiffness compared to untrained controls. The authors concluded that the shorter and stiffer tendons may favor rapid force production and reduce the risk of tendon fatigue injury, yet longer MRI T2* relaxation times of the free Achilles tendon could indicate accumulated damage. In their cross-sectional observations, Epro et al. showed that plantar flexor muscle strength and Achilles tendon stiffness are greater in the dominant leg of adult elite track and field jumpers, yet in a similar order of magnitude, suggesting a balanced adaptation of muscle and tendon. However, the same authors (Karamanidis and Epro) also presented evidence of imbalances when the adaptation of muscle and tendon was monitored over time. In a 1-year period of observation with measurements performed every 3–5 weeks, track and field jumpers had greater fluctuations of maximum tendon strain compared to untrained controls. Arampatzis et al. argued in their perspective paper that well-balanced muscle strength and tendon stiffness are important for healthy and effective MTU function and present a framework how it could be promoted by individualized muscle and tendon diagnostics and load prescriptions. Tailored loading for the tendon may also be a future approach in the treatment of tendinopathy. Wiesinger et al. provided evidence that tendon stiffness is reduced in patients with chronic patellar tendinopathy, while no significant differences were shown considering the capacity to store and release energy. Although the restoration of tendon mechanical properties may be targeted or monitored in therapeutic interventions, Kulig et al. called in their perspective article for a multilevel perspective in the treatment of tendinosis, highlighted the importance of the etiology of this condition and emphasized the necessity to also address the changes observed in the motor control system.

Considering muscle adaptation, Geremia et al. investigated changes in triceps surae muscle architecture in response to 12 weeks of eccentric training. All three muscles demonstrated an increase in muscle thickness and fascicle length, with the greatest changes of fascicle length in the soleus muscle. Moreover, Marzilger et al. showed on the vastus lateralis that, when time under tension and load magnitude is matched, the lengthening velocity during eccentric loading does not affect the extent of muscle hypertrophy. McMahon et al. shed light into the role of tumor necrosis factor alpha (TNFα) in the regulation of muscle mass during training and detraining. While in response to 8 weeks of resistance training no changes in systemic levels of TNFα were observed, the reduction of muscle mass during the following detraining period seemed to be mediated by the pro-inflammatory cytokine in healthy adults. In their review, Csapo et al. focused on the extracellular matrix of skeletal muscle, providing an overview about its composition, function, remodeling and adaptation. The authors concluded that its essential role is widely underestimated and may further be a target in the treatment of muscular and metabolic disorders consequent to aging or disease. The aging muscle was also subject of the original research article by Bruseghini et al. demonstrating that both aerobic high-intensity training and isoinertial resistance training reduce intermuscular adipose tissue and increase muscle volume of the quadriceps, yet only resistance training increases muscle physiological cross-sectional area, activation and strength in elderly men. The simplified method of muscle volume prediction for the triceps surae presented by Karamanidis et al. may be of use in future studies on the elderly and further provides evidence that age-induced atrophy may, in addition to its dimensions, change the muscle shape, at least in women as observed here. Singh et al. investigated the effects of a combined aerobic and resistance training, performed either one, two or three times a week, on walking economy of postmenopausal women. Regardless of training frequency, it seems that 16 weeks of combined training improves the ease of walking and economy. Finally, Venturelli et al. presented a case report of a young patient suggesting that resistance training can improve muscle strength and respiratory capacity in mitochondrial encephalomyopathy, lactic acidosis, and recurrent stroke-like episodes syndrome, a rare degenerative disease that is associated with massive deconditioning.

Our understanding on how muscle and tendon interact and how the MTU adapts to changes in mechanical loading as well as the influence of maturation, aging and pathology is continuously increasing. The present article collection sampled exciting new developments in these areas, yet it also points at many unanswered and new questions in this field. Those include the question of the optimization of muscle-tendon interaction via mechanical loading for increasing, maintaining or regaining movement performance. A better grasp of the complexity of three-dimensional muscle and tendon architecture remains one of the challenges to further research on MTU function. Likewise, more insight into the role and malleability of the extracellular matrix components is still critically missing. The processes of mechanotransduction in muscle and tendon are not well-understood, especially when it comes to potential changes due to aging or pathology. Advances in this area may contribute to increasing the effectiveness and efficiency of loading-based interventions and improve treatment success e.g., in tendinopathy or cerebral palsy. We would like to thank all contributors for their participation to this Research Topic and look forward to join our efforts in muscle and tendon research.

## Author Contributions

This manuscript was written by all authors. All authors have performed editorial reviewing tasks for this Research Topic.

## Conflict of Interest

The authors declare that the research was conducted in the absence of any commercial or financial relationships that could be construed as a potential conflict of interest.

